# Anti-Inflammatory Potential of Newly Synthesized 4-[(Butylsulfinyl)methyl]-1,2-benzenediol in Lipopolysaccharide-Stimulated BV2 Microglia

**DOI:** 10.3390/molecules191016609

**Published:** 2014-10-15

**Authors:** Guk-Heui Jo, Il-Whan Choi, Jin-Woo Jeong, Gi-Young Kim, Jinwoo Kim, Hongsuk Suh, Chung-Ho Ryu, Wun-Jae Kim, Yung Hyun Choi

**Affiliations:** 1 Department of Biochemistry, College of Korean Medicine, Dongeui University, Busan 614-052, Korea; E-Mail: cooki28@naver.com; 2 Department of Microbiology, College of Medicine, Inje University, Busan 608-756, Korea; E-Mail: cihima@inje.ac.kr; 3 Division of Applied Life Science (BK21 Plus), Gyeongsang National University, Jinju 660-701, Korea; E-Mail: immune-jeong@gnu.ac.kr; 4 Department of Marine Life Sciences, Jeju National University, Jeju 690-756, Korea; E-Mail: immunkim@cheju.ac.kr; 5 Department of Chemistry and Chemistry Institute for Functional Materials, Pusan National University, Busan 609-735, Korea; E-Mails: jinwoo@pusan.ac.kr (J.K.); hssuh@pusan.ac.kr (H.S.); 6 Division of Applied Life Science, Gyeongsang National University, Jinju 660-701, Korea; E-Mail: ryu@gnu.ac.kr; 7 Department of Urology, College of Medicine, Chungbuk National University, Cheongju 361-763, Korea; E-Mail: wjkim@chungbuk.ac.kr; 8 Anti-Aging Research Center & Blue-Bio Industry Regional Innovation Center, Dongeui University, Busan 614-714, Korea

**Keywords:** 4-[(butylsulfinyl)methyl]-1,2-benzenediol, BV2 microglia, anti-inflammation, NF-κB, MAPKs, PI3K/Akt

## Abstract

In this study, we investigated the anti-inflammatory effects of newly synthesized 4-[(butylsulfinyl)methyl]-1,2-benzenediol (SMBD) in lipopolysaccharide (LPS)-stimulated BV2 microglia and the subsequent signaling events. Following stimulation with LPS, elevated production of nitric oxide (NO) and prostaglandin E_2_ (PGE_2_) was detected in BV2 cells; however, SMBD pretreatment inhibited the production of NO and PGE_2_ through suppressing gene expression of inducible NO synthase (iNOS) and cyclooxygenase-2 (COX-2), respectively, at non-toxic concentrations. LPS-stimulated gene expression and production of interleukin (IL)-1β and tumor necrosis factor (TNF)-α were also significantly reduced by SMBD. The anti-inflammatory effects of SMBD were associated with suppression of LPS-induced nuclear translocation of nuclear factor-kappa B (NF-κB), and phosphorylation of mitogen-activated protein kinases (MAPKs) and Akt, a phosphatidylinositol 3-kinase (PI3K) downstream effector. Therefore, the present results demonstrate that SMBD down-regulates inflammatory gene expression by inhibiting the activation of NF-κB through interference with the activation of MAPKs and PI3K/Akt signaling. Taken together, our data suggest that SMBD may have potential to be developed into an effective anti-inflammatory agent.

## 1. Introduction

Although inflammation is part of the highly complex immune response to defend against harmful stimuli, chronic inflammatory processes are involved in the pathogenesis of common inflammation-associated diseases [1,2]. In particular, microglia are resident immune cells in the central nervous system (CNS) and their inflammatory response is primarily a host defense mechanism, but can also contribute to damage surrounding healthy neurons and lead to chronic progression of neurodegeneration [3,4]. In response to extracellular stimuli, microglia are activated and produce pro-inflammatory mediators, such NO and PGE_2_, and cytokines, as well as neurotoxic substances, which are thought to be responsible for neuronal injuries and diseases in the CNS [5,6]. In this context, NO is synthesized from L-arginine by iNOS [7]; and PGE_2_ is produced from arachidonic acid metabolites by COX-2 [8]. Additionally, IL-1β and TNF-α are the main inflammatory cytokines that are produced by activated microglia during CNS inflammation [9,10,11]. Collectively, the inhibition of neuroinflammation has been postulated as a putative target in the treatment of neurodegenerative diseases and control of microglial activation may alleviate the progression of neurodegeneration.

One of the favored models used to characterize microglia activation and to test the potential anti-inflammatory effects of candidate compounds is the stimulation of microglia by LPS obtained from Gram-negative bacteria [12,13]. LPS can bind to its cognate receptors on the microglial cell surface, leading to the activation of critical transcription factors such as NF-κB, which regulate various cellular genes involved in the inflammatory responses [14,15,16]. Furthermore, regulation of MAPKs, which in turn are classified into three components: extracellular signal-regulated kinase (ERK), c-Jun N-terminal kinase (JNK) and p38 MAPK, and PI3K/Akt pathways is also involved in alleviating inflammation through inducing the expression of inflammation-related genes [17,18,19]. Although, many studies have reported the potential anti-inflammatory effects of novel compounds, agents with proven efficacy and minimal toxicity are urgently required for the prevention and treatment of microglial activation.

**Figure 1:**
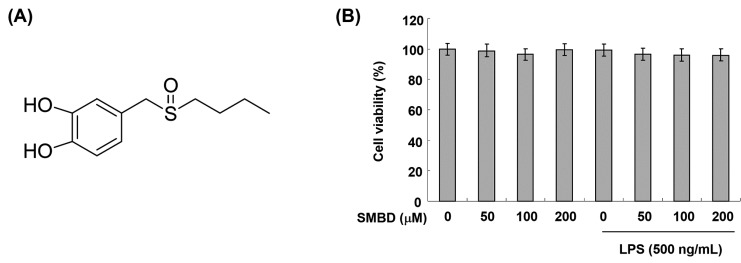
Effects of SMBD and LPS on the cell viability of BV2 microglia. (**A**) Chemical structure of SMBD. (**B**) Cells were treated with different concentrations of SMBD in the absence or presence of 500 ng/mL LPS. After 24 h, cell viability was assessed using an MTT reduction assay. The results are expressed as the percentage of surviving cells over control cells (without the addition of SMBD). Each value indicates the mean ± SEM of three independent experiments.

Several studies have reported that catechol, with the ortho-dihydroxyl group of the benzene ring, has radical scavenging and anti-inflammatory effects [20,21]. Furthermore, the dimethyl sulfoxide (DMSO), with two alkyl groups, has been reported to be an effective scavenger of reactive oxygen radicals [22,23,24]. Recently, Kim *et al.* [25] chemically synthesized 4-[(butylsulfinyl)methyl]-1,2-benzenediol (SMBD, Figure 1A) with a combination of catechol and DMSO, and found that SMBD shows antioxidant effects by lowering plasma lipid levels and decreasing aortic thickness, ROS generation and COX-2 protein expression in cholesterol diet-induced hypercholesterolemic rabbits.

In this study, as a part of our ongoing screening program to evaluate the anti-inflammatory potentials of novel synthetic compounds, we evaluated whether SMBD inhibits LPS-induced production of inflammatory mediators and cytokines using the murine BV2 microglial model. Our data indicate that SMBD exerts anti-inflammatory activity by reducing NO, PGE_2_, IL-1β, and TNF-α production in LPS-stimulated BV2 cells. We further investigated the effect of SMBD on the NF-κB, MAPKs and PI3K/Akt signaling pathways to clarify its inhibitory mechanisms. As a result of our findings, we suggest that SMBD is able to modulate the activities of inflammatory responses in activated microglia and SMBD may be a candidate for the treatment of various neurodegenerative disorders.

## 2. Results and Discussion

### 2.1. Effects of SMBD on the BV2 Cell Viability

To exclude the possibility that our results could be confounded by cytotoxic effects, we used the MTT assay to evaluate the viability of BV2 cells treated with various concentrations of SMBD. As shown in Figure 1B, cell viability was not significantly altered by treatment with SMBD concentrations up to 200 μM. Thus, in the following experiments, cells were treated with SMBD concentrations up to 200 μM.

### 2.2. SMBD Inhibits LPS-Induced NO Production and iNOS Expression in BV2 Cells

**Figure 2:**
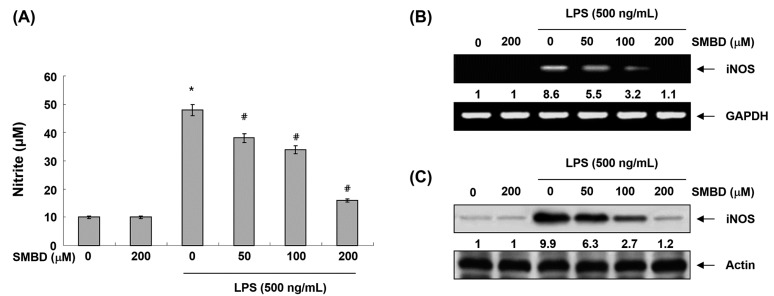
Effects of SMBD on LPS-induced NO production and iNOS expression in BV2 microglia. Cells were treated with the indicated concentrations of SMBD for 1 h and then incubated in the presence of LPS (500 ng/mL) for 24 h. (**A**) NO accumulation was measured using the Griess reaction in culture media. Data are presented as mean ± SEM, and are representative of triplicate experiments. * *p* < 0.05 compared to control (LPS-free); ^#^ *p* < 0.05 compared to cells cultured with 500 ng/mL LPS. The mRNA (**B**) and protein (**C**) levels of iNOS were determined by reverse transcriptase-polymerase chain reaction (RT-PCR) and Western blot analysis, respectively. The numbers represent the average densitometric analyses as compared with glyceraldehyde-3-phosphate dehydrogenase (GAPDH)(B) andactin (C) in, at a minimum, two or three different experiments.

To evaluate the anti-inflammatory activity of SMBD, we first investigated the effects of SMBD on the production of NO in LPS-treated BV2 cells. For this study, BV2 cells were stimulated with LPS for 24 h after being pretreated with various concentrations of SMBD for 1 h, and we measured the accumulation of nitrite in the culture media. Our data indicated that the SMBD concentration dependently inhibited nitrite levels in the conditioned media of LPS-treated BV2 cells (Figure 2A). We then tested whether SMBD-mediated inhibition of NO production was due to the suppression of iNOS expression. RT-PCR and Western blot analyses showed that, when LPS was added to BV2 cells, the expression of iNOS mRNA and protein increased up to approximately 8.6- and 9.9-fold over the basal level; however, SMBD suppressed the LPS-induced expression of iNOS mRNA in a concentration-dependent manner (Figure 2B,C), suggesting that the SMBD-mediated inhibition of NO production is mediated via the suppression of iNOS at the transcription level.

### 2.3. SMBD Inhibits LPS-Induced PGE_2_ Production and COX-2 Expression in BV2 Cells

**Figure 3:**
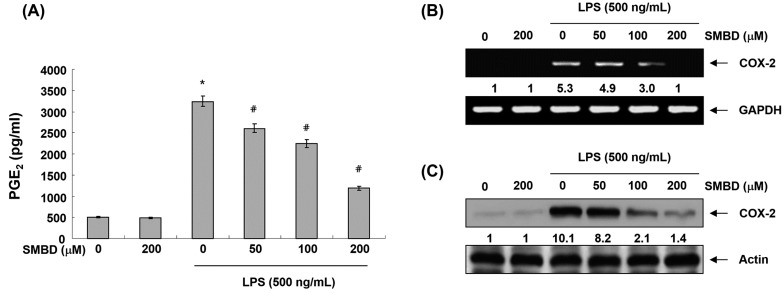
Effects of SMBD on LPS-induced PGE_2_ production and COX-2 expression in BV2 microglia. Cells were treated with the indicated concentrations of SMBD for 1 h and then incubated in the presence of LPS for 24 h. (**A**) PGE_2_ concentration was measured in culture media using a commercial ELISA kit. Data are presented as mean ± SEM, and are representative of triplicate experiments. * *p* < 0.05 compared to control (LPS-free); ^#^ *p* < 0.05 compared to cells cultured with LPS. The mRNA (**B**) and protein (**C**) levels of COX-2 were determined by RT-PCR and Western blot analysis, respectively. GAPDH and actin were used internal controls for the RT-PCR and Western blot assay, respectively. The numbers represent the average densitometric analyses as compared with GAPDH (B) andactin (C) in, at a minimum, two or three different experiments.

We next measured the accumulation of PGE_2_ in the culture media of SMBD-treated cells grown in the presence or absence of SMBD. Our results revealed that SMBD treatment inhibited LPS-induced PGE_2_ production in a concentration-dependent manner (Figure 3A). Furthermore, in response to LPS, the mRNA and proteins levels of COX-2 were markedly induced; however, pretreatment with SMBD inhibited these up-regulations in a concentration-dependent manner (Figure 3B,C), indicating that SMBD can down-regulate PGE_2_ production in LPS-stimulated BV2 cells via COX-2 expression inhibition at the transcriptional level.

### 2.4. SMBD Reduces LPS-Induced Production and Expression of Pro-Inflammatory Cytokines in BV2 Cells

**Figure 4:**
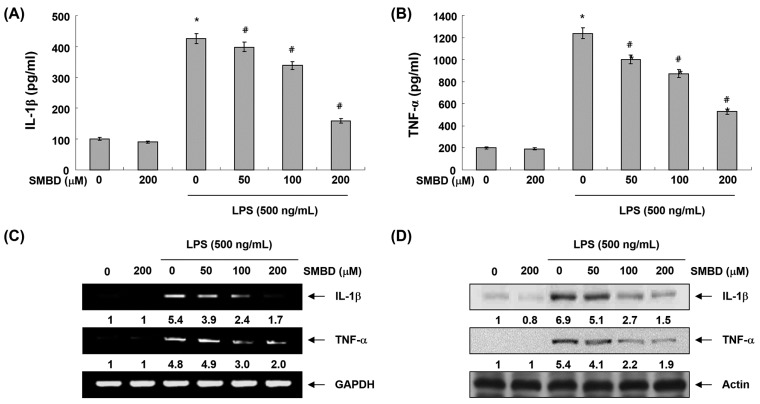
Effects SMBD on LPS-induced production and expression of IL-1β and TNF-α in BV2 microglia. BV2 cells were pretreated with various concentrations of SMBD for 1 h and then stimulated by LPS for 24 h. (**A** and **B**) Productions of IL-1β (**A**) and TNF-α (**B**) were measured using the corresponding ELISA kits. Data are presented as mean ± SEM, and are representative of triplicate experiments. * *p* < 0.05 compared to control (LPS-free); ^#^ *p* < 0.05 compared to cells cultured with 500 ng/mL LPS. The mRNA (**C**) and protein (**D**) levels of IL-1β and TNF-α were determined by RT-PCR and Western blot analysis, respectively. The numbers represent the average densitometric analyses as compared with GAPDH (C) and actin (D) in, at a minimum, two or three different experiments.

To further examine the inhibitory effect of SMBD on LPS-induced inflammatory responses, we measured pro-inflammatory cytokines, such as IL-1β and TNF-α, released into the culture medium using ELISA. As shown in Figure 4A,B, when BV2 cells were solely treated with SMBD, there were no significant changes in the production of either cytokine. However, both TNF-α and IL-1β levels increased in the culture media of LPS-stimulated BV2 cells, and these increases were significantly reduced by treatment with SMBD in a concentration-dependent manner. In a parallel experiment, RT-PCR and Western blot analyses were performed to determine whether SMBD inhibits the expression of IL-1β and TNF-α at the transcriptional or translational level. As shown in Figure 4C,D, treatment of BV2 cells with SMBD before LPS treatment resulted in a concentration-dependent decrease in the level of mRNA and protein of these cytokines, suggesting that SMBD-mediated inhibition of the tested cytokines may also be regulated at the transcriptional level.

### 2.5. SMBD Prevents the LPS-Induced Nuclear Translocation of NF-κB and Degradation of IκB-α in BV2 Cells

**Figure 5:**
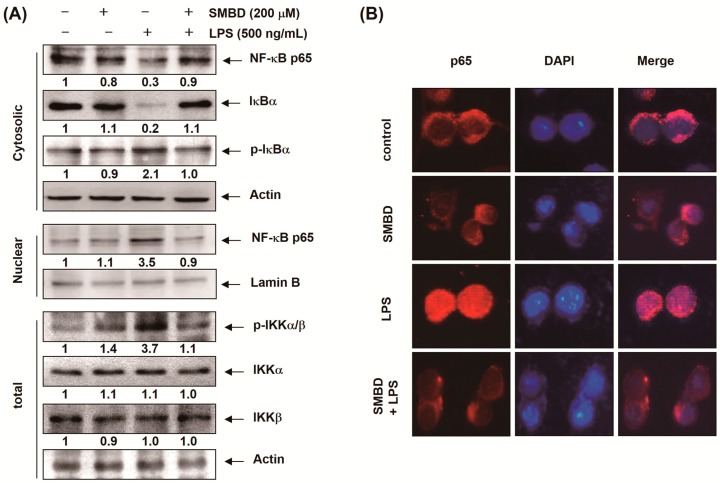
Effects of SMBD on the nuclear translocation of NF-κB and degradation of IκB-α in LPS-stimulated BV2 microglia. (**A**) BV2 cells were treated with 200 μM SMBD for 1 h before treatment with 500 ng/mL LPS for 30 min. Nuclear, cytosolic and total proteins were subjected to Western blot analyses with the indicated antibodies. Actin and lamin B were used as internal controls for the cytosolic and nuclear fractions, respectively. The numbers represent the average densitometric analyses as compared with actin and lamin B in, at a minimum, two or three different experiments. (**B**) Cells were pre-treated with 200 μM SMBD for 1 h prior to stimulation with 500 ng/mL LPS for 30 min. The cells were fixed, permeabilized, and incubated with a specific anti-NF-κB p65 antibody followed by an FITC-labeled anti-rabbit IgG antibody (red). Cell nuclei were visualized by DAPI staining (blue). The cells were visualized using a fluorescence microscope.

Since NF-κB is a major transcription factor that regulates the expression of various pro-inflammatory enzymes and cytokines, we investigated whether SMBD inhibits the release of NF-κB from IκB-α and/or the subsequent translocation of NF-κB from the cytosol to the nucleus. Western blot analyses using cytosolic and nuclear fractions showed that the accumulation of NF-κB p65 in the nucleus was markedly increased after treatment with LPS alone, concomitantly with the phosphorylation and degradation of IκB-α in cytosol; however, pretreatment with SMBD reversed these trends (Figure 5A). The immunofluorescence images also revealed that SMBD blocked the LPS-induced subsequent nuclear translocation of NF-κB (Figure 5B), indicating that SMBD prevented LPS-induced NF-κB translocation by attenuating the degradation of IκB-α. Next, we assessed whether SMDB regulates inhibitor of κB kinase (IKK) activation and expression, because IκBα is phosphorylated and degraded by IKK and NF-κB consequently releases from IκBα complex for unclear translocation. As presumed, LPS treatment led a significantly increase phosphorylation of IKK; however, SMDB significantly inhibited the phosphorylation of IKK, suggesting that SMDB regulates LPS-mediated IKK-IκBα-NF-κB circuit (Figure 5A).

### 2.6. SMBD Reduces the LPS-Induced Activation of MAPKs and PI3K/Akt in BV2 Cells

**Figure 6:**
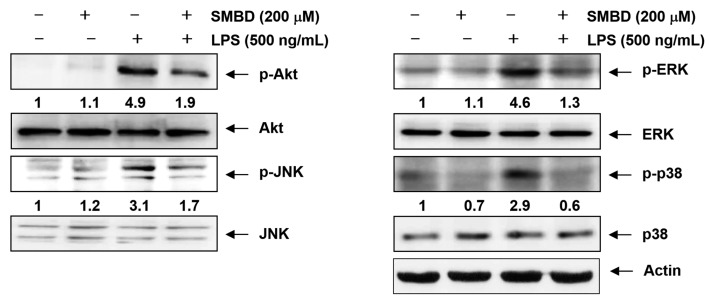
Effects of SMBD on the activation of MAPKs and PI3K/Akt pathways induced by LPS in BV2 microglia. BV2 cells were treated with 200 μM SMBD 1 h prior to treatment with 500 ng/mL LPS for 1 h. Whole cell lysates were prepared and were subjected to 10% SDS-polyacrylamide gels, followed by Western blot analysis using the indicated antibodies. Actin was used as internal control for the Western blot analysis.The numbers represent the average densitometric analyses as compared with actin in, at a minimum, two or three different experiments.

The MAPKs signaling cascade plays a pivotal role in the activation of microglial cells and PI3K’s involvement was also recently identified [26,27]. Therefore, to investigate whether SMBD regulates these pathways, we used Western blots to examine the phosphorylation of the three major MAPKs; JNK, ERK, and p38 MAPK, and Akt, a downstream effector of PI3K. As indicated in Figure 6, LPS treatment markedly increased the phosphorylation of Akt as well as the three MAPKs; however, pretreatment with SMBD markedly reduced the phosphorylation of these proteins in LPS-stimulated BV2 cells. Moreover, the total protein levels of Akt and MAPKs were not affected by LPS and SMBD treatment. Collectively, these results demonstrate that SMBD might block an LPS-induced expression of pro-inflammatory responses by inhibiting the MAPKs and PI3K/Akt signaling pathways in BV2 microglia.

### 2.7. Discussion

As a part of our search for novel biologically active substances for the prevention and treatment of inflammation-mediated diseases, the present study is designed to determine whether newly synthesized SMBD inhibits pro-inflammatory mediators and cytokines released from activated microglia, which play critical roles in various neurodegenerative diseases and have been associated with a variety of CNS diseases. Previous data found that the anti-oxidant effect of SMBD outstandingly prevents hyperchlolesterolemia. Currently, many anti-oxidants significantly attenuate LPS-induced pro-inflammatory gene expression. Nevertheless, it is little understood where SMBD down-regulates LPS-induced anti-inflammatory effects. Our present study showed that SMBD inhibits LPS-induced pro-inflammatory genes and mediators by suppression IKK-IκBα-NF-kB axis and MAPKs.

Microglia are activated in the injured brain to release various pro-inflammatory mediators, such as NO and PGE_2,_ and cytokines, including IL-1β and TNF-α, which may initiate or amplify the inflammatory responses in the CNS [5,6]. Moreover, high levels of NO and PGE_2_ produced by iNOS and COX-2, which are rapidly and highly expressed in LPS-activated microglia, have been shown to be cytotoxic to neuronal cells and contribute to the pathogenesis of septic shock [28,29]. Furthermore, the production of IL-1β and TNF-α is critically required for the synergistic induction of NO and PGE_2_ production in LPS-stimulated macroglia [9,11,30]. Therefore, candidate agents with ability to inhibit iNOS, COX-2, and these cytokines’ expression are potentially beneficial in the treatment of neuroinflammatory diseases associated with NO, PGE_2_ and cytokine overproduction. In this study, we have shown that SMBD significantly inhibited the NO and PGE_2_ synthesis by modulation of iNOS and COX-2 mRNA and protein in LPS-treated BV2 cells (Figure 2 and Figure 3). Moreover, observed inhibitions of the release of IL-1β and TNF-α might be attributed to the suppressions of IL-1β and TNF-α mRNA, and the subsequent reduction in their protein expressions (Figure 4). Therefore, the data indicated that SMBD exerts its anti-inflammatory effects via the repression of pro-inflammatory enzymes and cytokines at the transcriptional level. In addition, under the same experimental conditions, an MTT assay demonstrated that pretreatment of BV2 cells with SMBD in the presence or absence of LPS did not change cell growth (Figure 1B), indicating that the suppression of pro-inflammation mediators and cytokines by SMBD were not attributable to any nonspecific cytotoxic effect.

The transcription factor NF-κB is known to be a critical regulator of various genes involved in the regulation of many inflammatory enzymes and cytokines related to the inflammatory process [31,32]. In unstimulated cells, NF-κB is present in the cytosol as a homodimer or heterodimer, and is linked to the inhibitory protein, IκB. Following stimulation with LPS, NF-κB is activated via the activation of IκB-kinase complex, which then phosphorylates IκB, resulting in its subsequent proteasomal degradation as well as the release of NF-κB [16,33]. As a result, the free dimeric NF-κB translocates into the nucleus and binds to specific DNA sequences in the promoter regions of proinflammatory target genes for rapid transcription [14,15]. Recently, it revealed the importance of IKK as upstream regulator of the IκBα-NF-κB signaling pathway. IKK induces the phosphorylation and degradation of IκBα, and free NF-κB ultimately moves into the nucleus to accelerate pro-inflammatory gene expression. Therefore, IKK-IκBα-NF-κB is a good targeting axis for preventing inflammatory diseases.

The present results demonstrate that SMBD treatment attenuates the LPS-induced phosphorylation and degradation of IκB-α and translocation of the p65 subunit of NF-κB to the nucleus via suppression of IKK (Figure 5). Collectively, these results suggest that the inhibition of the NF-κB signaling pathway by SMBD results in the down-regulation of inflammatory mediators and cytokines, resulting in an anti-inflammatory effect in LPS-stimulated BV2 microglia.

Other downstream targets of LPS-induced inflammatory cascades in microglia are the MAPKs and PI3K/Akt signaling members. Previous studies have shown that activation of these molecules has a significant effect on the regulation of COX-2, iNOS, and inflammatory cytokine gene expression by controlling the activation of NF-κB in microglia [17,18,19]. Therefore, targeting the MAPKs and PI3K/Akt pathways is considered as an attractive therapeutic strategy for the development of anti-inflammatory drugs, and we investigated the effect of SMBD on LPS-stimulated phosphorylation of MAPKs and Akt in BV2 microglia (Figure 5). Our data demonstrated that SMBD markedly inhibited LPS-induced phosphorylation of three MAPKs as well as Akt. Thus, it is postulated that attenuation of the phosphorylation of MAPKs and Akt by SMBD might contribute to the inhibition of inflammatory reactions in LPS-stimulated BV2 microglia.

## 3. Experimental Section

### 3.1. Cell Culture and SMBD Treatment

BV2 murine microglial cells were provided by I.W. Choi (Inje Univ., Busan, Korea) and cultured in Dulbecco’s modified Eagle’s medium (DMEM, Gibco-BRL, Grand Island, NY, USA) supplemented with 10% fetal bovine serum (Gibco-BRL), 100 U/mL penicillin, and 100 μg/mL streptomycin, and 2 mM L-glutamine, at 37 °C in a 5% CO_2_-humidified air environment. SMBD was dissolved in phosphate-buffered saline (PBS) and diluted with DMEM to the desired concentration prior to use.

### 3.2. Cell Viability Assay

The rate of cell growth was determined colorimetrically using the MTT assay. Briefly, BV2 cells were incubated with the indicated concentrations of SMBD or 500 ng/mL of LPS (Sigma-Aldrich, St. Louis, MO, USA) alone, or pretreated with SMBD for 1 h before LPS. After 24 h, the medium was discarded and the cells were incubated with a 0.5 mg/mL of 3-(4,5-dimethylthiazol-2-yl)-2,5-diphenyltetrazolium bromide (MTT, Sigma-Aldrich, St. Louis, MO, USA) solution at 37 °C. Three hours later, the supernatants were removed and the formazan blue, which was formed in the cells, was dissolved with DMSO at room temperature for 5 min. Subsequently, absorbance was measured with an enzyme-linked immunosorbent assay (ELISA) plate reader (Dynatech Laboratories, Chantilly, VA, USA) set at an absorbance wavelength of 540 nm. The growth inhibition was assessed as the percent viability where vehicle-treated cells were taken as 100% viable.

### 3.3. Measurement of NO Concentration

NO production was assayed by measuring nitrite (a stable degradation product of NO) in supernatants of cultured BV2 cells using the Griess reagent (1% sulphanilamide, 01% *N*-(1-naphthyl)-ethylenediamine, 2% phosphoric acid, Sigma-Aldrich, St. Louis, MO, USA). Briefly, BV2 cells were cultured in 24-well plates. After reaching confluence, cells were pretreated with SMBD for 1 h as indicated, followed by 500 ng/mL of LPS, and then incubated in a humidified incubator at 37 °C for 24 h. The supernatant was collected, mixed with an equal volume of Griess reagent, and incubated for 10 min at room temperature. The absorbance measured at 540 nm in an ELISA plate reader was used as an indication of the nitrite concentration. Sodium nitrite (NaNO_2_) was used to produce a standard curve of nitrite concentrations [34].

### 3.4. Determination of PGE_2_, TNF-α, and IL-1β Production

To measure the quantity of PGE_2_, cells were incubated with different concentrations of SMBD in either the presence or absence of LPS for 24 h. Following the manufacturer’s instructions, a volume of 100 μL of culture medium supernatant was collected and the concentration (pg/mL) of PGE_2_ in the cell culture medium was calculated based on the concentrations of the standard solution using a PGE_2_ ELISA kit (Cayman Chemical Co., Ann Arbor, MI, USA). The levels of TNF-α and IL-1β in the supernatant were also measured by the ELISA kits (R&D Systems, Minneapolis, MN, USA) according to the manufacturer’s instructions.

### 3.5. RNA Isolation and RT-PCR

Total RNA was isolated with the RNAzol-B (Invitrogen Co., Carlsbad, CA, USA), according to the procedure reported by the manufacturer. Two micrograms of total RNA were reverse transcribed using M-MLV reverse transcriptase (Promega Co., Madison, WI, USA) following the manufacturer’s instructions. Single stranded cDNA was then amplified by PCR with specific primers of iNOS, COX-2, IL-1β, and TNF-α (Bioneer, Deajeon, Korea). After amplification, the PCR reactions were electrophoresed in 1% agarose gels and visualized by ethidium bromide (EtBr, Sigma-Aldrich, St. Louis, MO, USA) staining. In a parallel experiment, GAPDH was used as an internal control. The target bands were quantified using Image J software version 1.45. The intensity of a specific band was normalized by comparison with GAPDH and the results have been calculated and expressed as relative amounts.

### 3.6. Protein Extraction and Western Blot Analysis

For preparation of whole cell lysates, cells were washed with ice-cold phosphate buffered saline (PBS), and lysed in a lysis buffer [25 mM Tris-Cl (pH 7.5), 250 mM NaCl, 5 mM ethylene diaminetetra acetic acid (EDTA), 1% NP-40, 1 mM pheny-methylsulfonyl fluoride (PMSF), and 5 mM dithiothreitol (DTT)] for 1 h. In a parallel experiment, nuclear and cytosloic proteins were prepared using nuclear extraction reagents (Pierce, Rockford, IL, USA) according to the manufacturer’s protocol. The protein concentration was determined using a detergent-compatible protein assay from Bio-Rad Laboratories (Hercules, CA, USA), according to the instructions provided by the manufacturer. Equal amounts of protein were resolved by sodium dodecyl sulfate (SDS)-polyacrylamide gel electrophoresis, and transferred onto a nitrocellulose membrane (Schleicher & Schuell BioScience, Inc., Keene, NH, USA). The membrane was then washed with Tris-buffered saline (TBS, 10 mM Tris, 150 mM NaCl) containing 0.05% Tween 20 (TBST) and blocked in TBST containing 5% non-fat dried milk at room temperature. After 1 h, the membranes were further incubated with the desired antibodies of for 1 h, and then the membranes were continuously incubated with appropriate secondary antibodies coupled to horseradish peroxidase (Amersham Corp., Arlington Heights, IL, USA), and developed using the enhanced chemiluminescence (ECL) Western detection reagents (Amersham Corp., Arlington Heights, IL, USA). The immunoreactive bands were detected and exposed to X-ray film. Primary antibodies were purchased from BD Transduction Laboratories (Franklin Lakes, NJ, USA), Cayman Chemical (Ann Arbor, MI, USA), Santa Cruz Biotechnology, Inc. (Santa Cruz, CA, USA) and Cell Signaling Technology, Inc. (Danvers, MA, USA) (Table 1). The target bands were quantified using Image J software version 1.45. The intensity of a specific protein band was normalized by comparison with actin and the results have been calculated and expressed as relative amounts.

**Table 1:** List of antibodies used in this study.

Antibody	Dilution	Product No.	Species of Origin and Supplier
iNOS	0.3888889	SC-509	rabbit polyclonal, BD Transduction Laboratories
COX-2	0.7361111	160126	rabbit polyclonal, Cayman Chemical
IL-1β	0.3888889	SC-7884	rabbit polyclonal, Santa Cruz Biotechnology, Inc.
TNF-α	0.3888889	#3707	rabbit polyclonal, Cell Signaling Technology, Inc.
NF-κB p65	0.3888889	SC-109	rabbit polyclonal, Santa Cruz Biotechnology, Inc.
IκB-α	0.3888889	SC-847	rabbit polyclonal, Santa Cruz Biotechnology, Inc.
Lamin B	1.4305556	SC-6216	goat polyclonal, Santa Cruz Biotechnology, Inc.
p-IKKα/β	0.3888889	SC-23470(R)	rabbit polyclonal, Santa Cruz Biotechnology, Inc.
IKK-α	0.7361111	SC-7606	mouse monoclonal, Santa Cruz Biotechnology, Inc.
IKK-β	0.3888889	SC-34673	goat polyclonal, Santa Cruz Biotechnology, Inc.
Akt	0.3888889	SC-8312	rabbit polyclonal, Santa Cruz Biotechnology, Inc.
p-Akt	0.3888889	SC-101629	rabbit polyclonal, Santa Cruz Biotechnology, Inc.
JNK	0.7361111	#9252S	rabbit polyclonal, Cell Signaling Technology, Inc.
p-JNK	0.3888889	#9255	mouse monoclonal, Cell Signaling Technology, Inc.
ERK	1:1,000	SC-154	rabbit polyclonal, Santa Cruz Biotechnology, Inc.
p-ERK	0.3888889	#9106S	mouse monoclonal, Cell Signaling Technology, Inc.
p38	1:1,000	SC-535	rabbit polyclonal, Santa Cruz Biotechnology, Inc.
p-p38	0.3888889	#9211S	rabbit polyclonal, Cell Signaling Technology, Inc.
Actin	1:1,000	SC-1615	goat polyclonal, Santa Cruz Biotechnology, Inc.

### 3.7. Immunofluorescence Staining for NF-κB p65

Cells were cultured on glass coverslips in 6-well plates, treated with 200 μM SMBD for 1 h, and then incubated in the presence of LPS (500 ng/mL) for 30 min. The cells were washed twice with PBS, fixed with 4% paraformaldehyde in PBS for 10 min at room temperature and permeabilized with 100% methanol for 10 min at 20 °C. Following two more washes with PBS, the cells were incubated overnight at 4 °C with an anti-NF-κB p65 antibody followed by a 1 h incubation with FITC-conjugated donkey anti-rabbit IgG (Sigma-Aldrich, St. Louis, MO, USA). After washing with PBS, the nuclei were stained with 4'6-diamidino-2-phenylindole (DAPI, Sigma-Aldrich, St. Louis, MO, USA) and the fluorescence was visualized using a fluorescence microscope (Carl Zeiss, Jena, Germany).

### 3.8. Statistical Analysis

Results are expressed as the mean ± the standard error of the mean (SEM) from three independent experiments. One-way analysis of variance (ANOVA) followed by, when appropriate, Bonferroni’s multiple range test, was used to determine the statistical significance of the difference between means. A *p*-value of less than 0.05 was taken as statistical significance.

## 4. Conclusions

In summary, our study revealed that SMBD is suitable for reducing the LPS-induced production of pro-inflammatory mediators and cytokines through the suppression of transcription of those genes. The inhibitory effect of SMBD was mediated by an inhibition of NF-κB via IKK-dependent IκBα degradation, which was associated with the inactivation of multiple MAPKs and PI3K/Akt signaling pathways in activated microglia. Based on these findings, we suggest that SMBD may block the LPS-induced initiation of intracellular inflammatory signaling cascades, and SMBD could be a candidate for the treatment of various inflammatory diseases that are associated with the overactivation of microglia.
